# Developing a therapeutic app based on the emotional Stroop task for objective discovery of daily life issues for people with ADHD

**DOI:** 10.3389/fpsyg.2025.1502914

**Published:** 2025-03-12

**Authors:** S. Schoenmakers, S. H. Bos, W. A. Ijsselsteijn

**Affiliations:** Eindhoven University of Technology, Human Technology Interaction, Eindhoven, Netherlands

**Keywords:** ADHD, emotional Stroop test, attentional control, emotional interference, diagnostic measurement, daily life issues, therapy

## Abstract

Pinpointing the most urgent problem to start treatment on in therapy for people with ADHD is a subjective and time-consuming process. To improve this process, we designed a proof-of-concept for an application that can identify daily life issues that cause negative mental load. Through several modified emotional Stroop-tasks, we show that people with ADHD respond slower to negative emotions and daily life issues related to ADHD, compared to neurotypicals. Negative emotions and social issues were especially prevalent in the ADHD participants. The application highlighted two to five issues per participant. This could indicate that these topics cause the highest mental load in the participant, and need attention first from a therapist. Using this application in a therapeutic context could deliver a more objective, personalized, traceable and efficient therapy for daily-life issues in ADHD.

## 1 Introduction

Attention-deficit hyperactivity disorder (ADHD) is a common neurodevelopmental disorder, which has considerable impact on the daily lives of many adults. The symptoms of ADHD can affect work performance, relationships with partners, family and friends, reliability and self-image or the ability to relax sufficiently (Adler and Cohen, 2004; Adler and Shaw, 2011; American Psychiatric Association, 2013; Kooij and Francken, 2010).

Patients with ADHD often experience difficulties in executive functioning and emotion regulation. This impacts a variety of functions such as inhibitory control (impulse control), working memory, shifting attention, control of attention and cognitive flexibility, response inhibition, reward sensitivity, temporal information processing, timing issues, speech functions, language functions, motor control problems, memory span, processing speed, arousal/activation, reaction time variability, and regulation of mood and emotion (Diamond, 2013; Faraone et al., 2015; Johnson, 2012). Because of the disruption of all these functions, ADHD can have serious consequences in daily life (Adler and Shaw, 2011; Faraone et al., 2015).

Currently, diagnosis of ADHD is an exhaustive process, consisting of many subjective components, such as interviews and questionnaires, to examine cognitive dysfunctionalities. After the diagnosis, another analysis phase follows to find out which issues a person with ADHD struggles with the most to start treatment on aspects that will be most helpful.

Due to the heterogeneity of the disorder, every patient has a very specific set of issues, which also changes over the course of their life depending on which life phase they are in.

A digital application could support the therapeutic process by guiding the patient and therapist quickly in the right direction, and save three up to 5 h out of a 20-h-therapy budget in Netherlands, leaving more time for actual treatment of the patient because of technological support to the therapist.

With this study we intend to develop an application to support the patient and therapist in identifying the most important issues in the patient’s daily life in a quick and objective fashion.

### 1.1 Modified Stroop task

The original Stroop task was designed to measure interference effects on attention (Stroop, 1935). Yet, the Stroop task can be modified to assess cognitive information overload or informational biases (MacLeod, 1991). An emotional stimulus has a stronger bottom-up signal than a neutral stimulus, thus interfering more with this Stroop task. Due to stronger interference, the reaction time of naming the inkcolor is longer. This application of the Stroop task is also known as the “Emotional Stroop Task” or “affective Stroop task” (MacLeod, 1991; Raschle et al., 2017; Williams et al., 1996).

Williams et al. (1996) studied various applications of the emotional and modified Stroop Task: anxiety, post-traumatic stress disorder (PTSD), panic disorder, obsessive-compulsive disorder (OCD) and social, spider and snake phobia’s and depression. They found patients to be slower to name the color of a word associated with concerns relevant to their clinical condition. A more recent example is the study by Kramer and Goldman (2003), in which they modified the Stroop task to contain words associated with substance abuse. They found response latencies to be longer for participants struggling with substance abuse.

A modified Stroop task has also been effectively applied to mere association. Sparrow et al. (2011) modified the original Stroop task by adjusting the words from colors to company related and neutral words. The results showed that the reaction times for company related words increased when a task needed to be performed that the company could help you with. This seems to imply that when you set a context, words that are related and common for that context show increased reaction times.

### 1.2 Emotional dysregulation in ADHD

One mayor result of the cognitive problems in ADHD, is difficulties with emotion and self-regulation of emotion (Shaw et al., 2014). Self-regulation of emotion is the ability for inhibition while being in an emotional state, which allows to delay responding to emotional events, or to respond with moderation. Several studies indicate a significant link between ADHD and comorbid symptoms of emotional dysregulation (Anastopoulos et al., 2011; Barkley, 2006; Graziano and Garcia, 2016; Karalunas et al., 2020; Landis et al., 2020; Lugo-Candelas et al., 2017; Strine et al., 2006), some even suggest emotional dysregulation to be a core component of ADHD (Shaw et al., 2014).

Often go/no-go tasks or n-back tasks are used to study emotional dysregulation in ADHD, like in Marx et al. (2011) showing lowered performance for people with ADHD when negative emotional stimuli were involved. Similarly, children with ADHD responded slower when negative emotional distractors were present in Villemonteix et al. (2017). In contrast, Karalunas et al. (2020) illustrate greater early attention capture by positive stimuli. In many studies it is shown that emotional interference in ADHD seems to affect response time rather than response accuracy (López-Martín et al., 2013; Marx et al., 2011; Villemonteix et al., 2017).

The emotional dysregulation in people with ADHD could be leveraged in the modified Stroop tasks since it is expected that slowed responses due to stimuli that have a negative connotation to the participant will provoke an even greater slow down of the response than in neurotypicals as in Williams et al. (1996).

### 1.3 Modified Stroop task for ADHD

In the meta-analyses and reviews across *>*20 studies by Lansbergen et al. (2007); Schwartz and Verhaeghen (2008), ADHD individuals were found to be up to 1.14 times slower than age-matched controls in both the color and the color-word condition for the traditional Stroop task. Both meta-analyses reveal more interference for the ADHD groups relative to the control groups.

Several studies have recently shown that the original Stroop task can be used to diagnose ADHD with a clear trend towards making ADHD measurable with portable or wearable devices that can be administered in the home setting. For instance, frontal EEG measures showed that asymmetries in the frontal signals and absolute power in the delta, and beta band during the execution of the Stroop task had diagnostic value for ADHD (Yoon et al., 2023). fNIRS measures and machine learning methods have been used in combination with a Stroop or Reverse Stroop Task to reach very high diagnostic levels for ADHD vs. neurotypicals (Maniruzzaman et al., 2024; Yang et al., 2023; Yasumura et al., 2020). Moreover, eyetracking during a Stroop task showed that longer saccade latencies, more saccades and shorter fixation periods could diagnose ADHD (Yoo et al., 2024). Also, a wearable device that passively collected data during a Stroop task on heart rate variability, electrodermal activity, and skin temperature demonstrated that physiological data during the Stroop task has diagnostic value for ADHD vs. neurotypicals (Andrikopoulos et al., 2024). Furthermore, motion tracking with a Kinect during a Stroop task indicated promising results for ADHD diagnosis (Li et al., 2024). However so far, we were unable to find similar results for a modified Stroop tasks in ADHD.

Many task in ADHD that were performed up until now, are only measured during non-emotional tasks (Karalunas et al., 2020), possibly because emotional stimuli will have unexpected influence on the performance of people with ADHD. However, in this study we actually want to benefit from both deficits in attentional control and emotional dysregulation in people with ADHD by designing a Stroop task that triggers their issues, whereby we intend to measure which issues in particular cause a lot of attention control deficits in a specific person.

Our research aim is to develop a easy-to-use task that can be used in a therapeutic or home-setting to investigate which problems are most prevalent at a certain time-point for a person with ADHD. To accomplish this, we have defined three research questions:

*Research question 1:* Can we create a Modified Stroop Task for ADHD personality traits that distinguishes people with ADHD from neurotypicals? This question is formulated to validate if personality traits related to ADHD envoke a latency in responses in people with ADHD based on association with their subjective feelings about personality traits.

*Research question 2:* Can we create a Modified Emotional Stroop task that distinguishes between ADHD and neurotypical people? The second question is formulated to validate if strong emotional words with positive or negative valence envoke latency in responses for people with ADHD.

*Research question 3:* Can we create a Stroop task for daily life domains in ADHD that can distinguish people with ADHD from neurotypicals? The final question is the envisioned extension of the theory to a new measuring instrument that could measure daily life issues that are most emotionally laden for people with ADHD.

## 2 Method

### 2.1 Experimental design

The experiment had a mixed design with 2 between factors (ADHD or not) and 3 within factors (responses to ADHD personality traits, responses to emotions, responses to ADHD daily life issues).

All participants completed eight modified Stroop tasks. The first section measured responses to ADHD personality traits, the second section measured responses to emotion stimuli, and the third section measured responses to five daily life domains.

The categories of words in the tasks were: related stimuli, unrelated stimuli and distraction stimuli. Except for the second section on emotion, which contained words from slightly different categories: positive, neutral, negative emotions and distraction stimuli. The dependent variable is reaction time. The independent variables are the categories, and whether the participant had an ADHD diagnosis or not.

After completing the experiment, participants were asked to fill in an online survey including demographics, screening for ADHD, other related mental health issues, and color blindness.

### 2.2 Participants

136 subjects (70 male, 65 female) in the age range between 18 and 81 (M = 30.77, SD = 14.44) participated. 47 participants were diagnosed with ADHD in the past. 85 participants had no diagnosis in ADHD, 16 of these participants would describe themselves as someone that could possibly have undiagnosed ADHD and were therefore excluded from our analysis. All participant signed an informed consent form and a privacy policy that was approved by the ethical review board of Eindhoven University of Technology. Participants were offered a monetary compensation for their participation.

### 2.3 Experimental stimuli

The words in the sections on ADHD and the five daily life domains were based on the DIVA (Kooij and Francken, 2010), which is a guideline for diagnostic interviews to investigate the presence of ADHD. The stimuli in these (sub)sections are based on the bulletpoints describing possible difficulties with those daily life domains and complemented based on conversations with people diagnosed with ADHD in a pilot. The stimulus set was not meant to be exhaustive, but was aimed to contain a set of common issues. The main focus here was on obtaining a well balanced set of words of similar length and amount of syllables. The five questions asked right before each subsection of the daily life domains are based on the DIVA as well (Kooij and Francken, 2010).

The words from the section on emotional interference were selected from validated datasets (Moors et al., 2013; Verheyen et al., 2020). To further validate this, each participant was asked to rate each emotion stimulus on valence in our study.

The distraction stimuli were obtained from two datasets containing Dutch words (Moors et al., 2013; Verheyen et al., 2020). These datasets contained rankings on valence, arousal and dominance for each word. Stimuli were selected on valence scores of 3.5–4.5 on a scale from one to seven, and arousal and dominance scores below average (<4.0). The stimuli were shown in psuedo-randomized order, with each word presented once in red and once in blue. Three researchers critically revised all sets of stimuli. The final set of stimuli that was used for the experiment can be found in Supplementary A.

### 2.4 Survey and screeners

Inquisit was used to run the experiment and automatically guided the participant to the Limesurvey for the follow-up survey. In the survey, first some demographic questions were asked and since participants had to distinguish red from blue stimuli, all participants were screened for color blindness with The Ishihara Test for Color Blindness (Ishihara, 1987).

Secondly, we inquired whether participants had officially been diagnosed with ADHD. If the participant would suspect undiagnosed ADHD, they had option to indicate this in a follow-up question. All participants were asked if they used any medication related to attention or emotion regulation. Furthermore, all participants were screened for ADHD with the ADHD Screener for Adults (Kooij and Buitelaar, 1997).

As neurodevelopmental disorders often co-occur and since they can influence subjective and objective measurement of symptoms (Jang et al., 2013; Murray, 2010; Sinzig et al., 2009), all participants were asked for diagnoses in comorbid disorders, such as ASD, dyslexia, other learning disorders, motoric disorders or anxiety disorders. They were also screened for all these mental disorders by use of the DSM-5 Self-Rated Level 1 Cross-Cutting Symptom Measure for Adults (American Psychiatric Association, 2013).

The last part of the survey asked participants to rate all the emotion words from the section on emotion on a valence scale from −3 to +3, in order to be able to analyze results on the emotions more accurately and individually.

### 2.5 Procedure

In inquisit, participants were asked to complete eight rounds of a modified Stroop task, where they had to name the ink color of a stimulus. They received instructions via the Inquisit WebPlayer. After successfully completing a test round, participants were asked to respond to stimuli that were personality traits of people with ADHD (the ADHD personality trait Stroop task). The second section contained stimuli on positive, negative and neutral emotions (the emotion Stroop task). The third section (the ADHD daily life Stroop tasks) consisted of five subsections where they had to answer a short open question on a daily life domain to prime them on the domain. The five sections were (1) work and study, (2) relationships and family, (3) social connections, (4) hobbies and free time, and (5) self-image and confidence. Each question was followed by a short modified Stroop task with stimuli applicable to that specific domain. After completing the experiment, participants were guided to a survey in Limesurvey for demographics and medical information.

### 2.6 Data analyses

All data analyses were carried out in STATA (StataCorp., 2021).

Variable average latency: Average latencies per stimulus were calculated per participant (*𝑙𝑎𝑡𝑟𝑒𝑑* + *𝑙𝑎𝑡𝑏𝑙𝑢𝑒* /2) to get a mean estimate per word in the datasets. For outlier removal, nine extreme values with latencies larger than 2,000 ms were detected, and removed. Furthermore, two observations with smaller latencies than 100 ms were dropped. Standardized scores (z-scores) of the response latency were calculated per word across participant, as the mean response latency varies a lot between participants.

Variable percentage of words of interest: For the statistical analysis, the number of “words of interest” per category and per participant was calculated. For a value to be a word of interest, the response latency had to be above a certain z-score. The analysis was performed with four different cut-off values: z-scores of 0.5, 1.0, 1.5 and 2.0. Based on the ADHD personality trait Stroop task we selected a z-score of 1.5 for the daily life tasks, since this led to a reasonable amount of words of interest per participant, but in essence this cut-off is arbitrary and can be set by the therapist.

The “percentage words of interest” was calculated by dividing the amount of words of interest by the total amount of words in the corresponding category.

*Research question 1:* Modified Stroop Task for ADHD traits: A two-way ANOVA was performed to analyze the effect of having ADHD and word category (personality traits related to ADHD, not related to ADHD, distraction words) on the mean percentage of number of words of interest between word categories.

*Research question 2:* Emotional interference in ADHD: A moderated regression analysis was performed to test if the valence of an emotion word influences response latency, and whether this is moderated by ADHD characteristics.

*Research question 3:* Stroop task for daily life domains in ADHD: A logistic regression was performed to see if the ADHD diagnosis can be predicted from the words of interest in the five daily life domains with a Wald Chi-Squared Test. Variables in the model are the mean percentage of words of interest between word categories (related, unrelated, distraction), across diagnosis (ADHD versus controls), and over the five domains of daily life (work, relations, social, free time, self-image). A second model was created with the ADHD screener added in order to see how our daily life domain compares to a commonly used ADHD screener.

## 3 Results

### 3.1 Research question 1: modified Stroop task, ADHD personality traits

**Figure 1:**
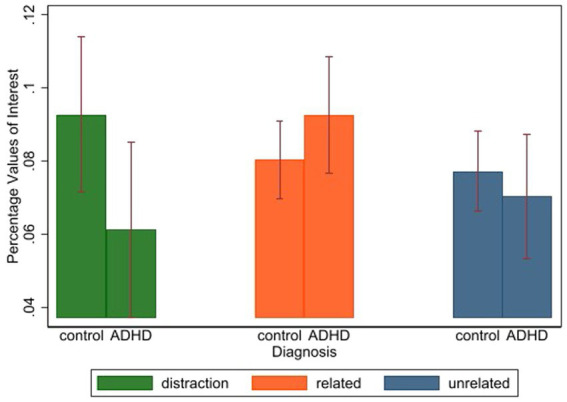
Visualization results for threshold z = 1.5. This figure visualizes the interaction between participants with or without ADHD for different word categories. On the y-axes the percentage words of interest is plotted.

The ANOVA for ADHD personality traits Stroop task showed that there is a significant difference in the number of words of interest between diagnosed ADHD participants and neurotypicals for thresholds of z = 0.5 or z = 1.0 (*F*(1,396) = 4.10, *p* = 0.045, *𝜂*^2^ = 0.012 and F(1,396) = 4.04, *p* = 0.044, $\eta_{p}^{2}$ = 0.010). A detailed overview with the test statistics can be found in Table 1. The only statistically significant interaction effect (*F*(2,396) = 3.41, *p* = 0.034, $\eta_{p}^{2}$ = 0.015) between ADHD diagnosis and stimulus category on the relative number of words of interest was observed at a threshold of z = 1.5. This interaction effect is visualized in Figure 1. When we looked at the interaction effect at z = 1.5, we saw that participants with ADHD responded slower to ADHD related words than control, yet for unrelated-or distraction-words participants with ADHD tended to be faster than control participants.

**Table 1:** Results ANOVA.

Threshold	Two-way ANOVA	*df*	*F*	$\eta_{p}^{2}$
z *>* 0.5	*𝑅*^2^ = 0.020			0.020
	Diagnosis	1	4.10*	0.012
	Category	2	0.37	0.001
	Diagnosis#category	2	1.51	0.008
z *>* 1.0	*𝑅*^2^ = 0.030			0.030
	Diagnosis	1	4.04*	0.010
	Category	2	1.03	0.007
	Diagnosis#category	2	2.15	0.011
z *>* 1.5	*𝑅*^2^ = 0.022			0.022
	Diagnosis	1	1.92	0.004
	Category	2	1.09	0.006
	Diagnosis#category	2	3.41*	0.015
z *>* 2.0	*𝑅*^2^ = 0.015			0.015
	Diagnosis	1	0.63	0.003
	Category	2	2.68	0.010
	Diagnosis#category	2	1.19	0.005

ANOVA was performed for different z-scores. The *𝑅*^2^ is the percentage of the data that is predicted by the current model, with an effect size of $\eta_{p}^{2}$. Then, the *F*-statistic and degrees of freedom (*df*) are presented for the variable percentage word of interest. These results are presented for difference in mean percentage between diagnoses (ADHD versus control), between categories (related, unrelated, distraction) and for the interaction between diagnosis and category. Significant effects are marked with an asterisk (∗ for *p* < 0.05, ∗∗ for *p* < 0.01, ∗∗∗ for *p* < 0.001).

### 3.2 Research question 2: emotional interference in ADHD

**Figure 2:**
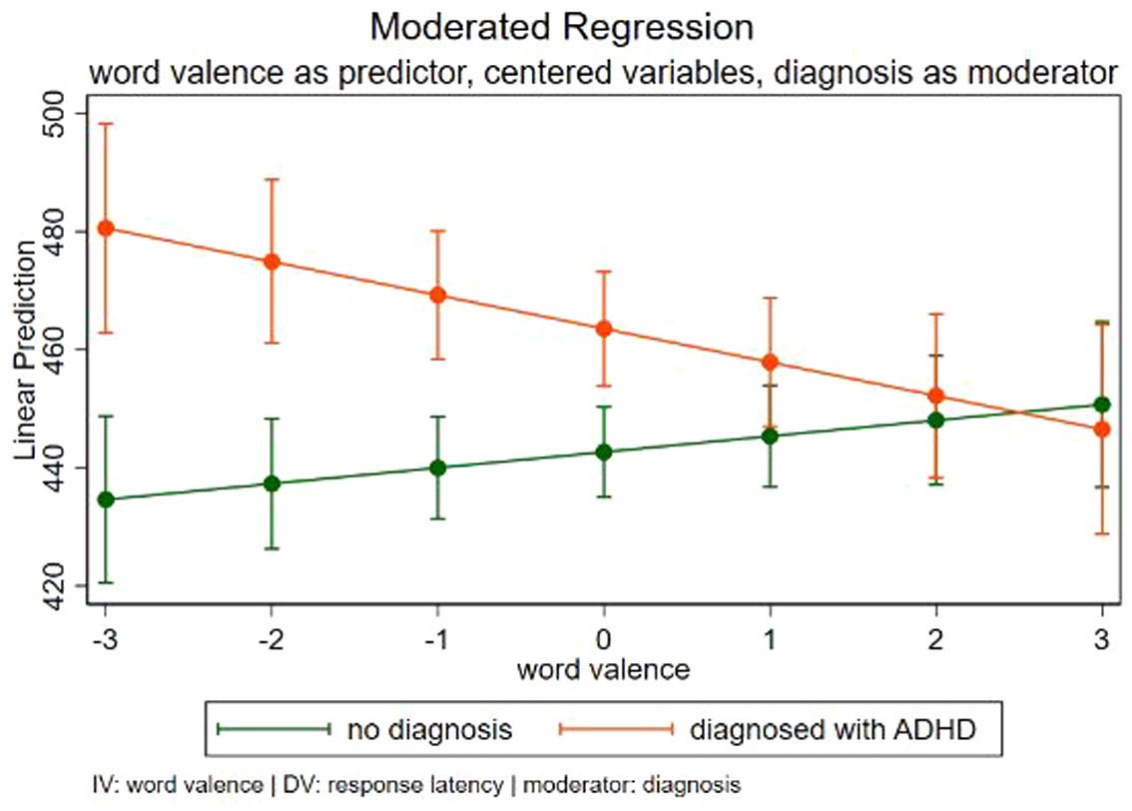
Plot for interaction effect of modified Stroop task responses for emotion words with negative to positive valence. Green visualizes the control group. Orange shows the responses for the group of participants with ADHD.

The moderated regression model is statistically significant (*F*(5, 3,583) = 11.89, *p <* 0.001). For the regression analysis, the interaction between word valence and ADHD diagnosis was significant on response latency (*F*(5, 3,583) = 11.89, *p <* 0.001, b = −8.365, t(115.78) = −2.65, *p* = 0.008 with $\eta_{p}^{2}$ = 0.016), where the more negative the valence of the emotion word, the slower the response of people with ADHD and the faster the response of controls. This is reported in Table 2 and in Figure 2 the results are visualized.

**Table 2:** Results moderated regression.

Moderated regression model	Coefficient	*t*	$\eta_{p}^{2}$
Model, *𝑅*^2^ = 0.016, *F*(3,3,153) = 5.94***			0.006
Valence	2.68	1.34	≤0.001
Diagnosis	20.89	3.32***	0.003
Valence#diagnosis	−8.36	−2.59**	0.002

For the model, the *R*^2^ and test statistic with degrees of freedom (*F*(*df*)) are reported. Per variable the regression coefficient, test statistic (*t*) and effect size ($\eta_{p}^{2}$) are reported. Significant effects are marked with an asterisk (* for *p* < 0.05, ** for *p* < 0.01, *** for *p* < 0.001).

### 3.3 Research question 3: Stroop task for daily life domains in ADHD

The logistic regression results (3) showed that Model 1 has a significant model fit (*Chi*^2^ (6) = 13.48, p = 0.03). The percentage of correct diagnoses increased by 5%. The coefficient on the variable for percentage words of interest in the social connections domain were statistically significant (z = −0.48, *p <* 0.01). Yet, the four other domains did not show significant results. Model 2, with the added ADHD screener, showed that the screener is a significant contribution (z = 4.85, *p* ≤ 0.001). By adding the ADHD screener, the sensitivity of the model increases by 38.3%, yet the specificity stays the same. This is reported in Table 3. With a z-score of 1.5, a mean 3.38 words (std = 1.04) out of a total of 50 words were identified per individual participant as the most important topics with long reaction times.

**Table 3:** Logistic regression results.

	Model 1			Model 2		
Variable	Odds ratio	Coef.	Wald’s z	Odds ratio	Coef.	Wald’s z
LR *Chi*^2^ (*df*)	13.48[6]	*p* = 0.036		43.67[7]	*p* ≤ 0.001	
Pseudo *𝑅*^2^	0.078			0.252		
Perc ADHD	1583.59	7.367	1.90	13350.5	9.500	2.08*
Perc work	3.52	1.259	0.46	0.848	−0.165	−0.05
Perc relationships	0.052	−2.949	−1.05	0.021	−3.880	−1.21
Perc social	0.0001	−8.873	−2.90 **	0.0002	−8.535	−2.51 *
Perc hobby	0.350	−1.049	−0.42	0.820	−0.198	−0.07
Perc self image	0.300	−1.205	−0.48	0.147	−1.920	−0.69
ADHD screener				1.275	0.050	4.85***
Sensitivity	27.66%			65.96%		
Specificity	88.51%			88.51%		
Correctly classified	67.16%			80.60%		

The dependent variable is ADHD diagnosis. The variable perc entails the percentage of values that are words of interest (*Z*-score *>* 1.5) in that category. The Wald statistics are distributed chi-square with 1 degree of freedom. Significant effects are marked with an asterisk (* indicates that the coefficient is statistically significant at the *p* < 0.05 level, ** for *p* < 0.01, *** for *p* < 0.001).

## 4 Discussion

The main research aim was to design a modified Stroop task to support therapists that help people with ADHD. The modified Stroop task is assumed to result in longer reaction times when a participant experiences interference of emotions and attentional control (Williams et al., 1996). We researched this application to daily life issues in adults with ADHD.

### 4.1 Research question 1: modified Stroop task with ADHD traits

In this first section, we tested the differences in response times on a modified Stroop task with ADHD related words, ADHD unrelated words and distraction words, between people with ADHD and controls. We found that participants with ADHD had longer reaction times than controls with words that referred to ADHD related personality traits in contrast to unrelated and distraction words. This result is in line with the results from Kramer and Goldman (2003); Williams et al. (1996), who investigated a modified Stroop task in other mental diagnoses. The ADHD personality traits that were shown in this task might be seen as negative traits related to their diagnosis and could therefore interfere with response time, as was the case in the study by Williams et al. (1996), where patients were often slower to name the color of a word associated with concerns relevant to their clinical condition. However, the ADHD personality traits do not have to be seen as negative traits, they could merely be associated with the participant’s own traits following the study by Sparrow et al. (2011).

### 4.2 Research question 2: emotional interference in ADHD

Secondly, we tested the effect of emotional interference on attentional control with a modified Stroop task that contained emotion words (with negative, neutral, or positive valence). The results show that subjects with ADHD have significantly larger response times for negative words than controls. The difference between ADHD and neurotypicals decreases as the stimuli become more neutral or positive, to the point where we measured no difference for positive emotion words for people with ADHD and controls.

The difference in negative stimuli between ADHD and control subjects supports the theory that people with ADHD have a strong emotional dysregulation (Anastopoulos et al., 2011; Barkley, 2006; Graziano and Garcia, 2016; Karalunas et al., 2020; Landis et al., 2020; Lugo-Candelas et al., 2017; Shaw et al., 2014; Strine et al., 2006). Because of the finding by Karalunas et al. (2020), unanticipated was the finding that there was no difference found in response times for positive stimuli, implying that this emotional dysregulation was only present for negative emotions in our study. In a meta-analysis by Graziano and Garcia (2016) it was reported that children with ADHD are reactive to both positive and negative emotions, yet rarely are positive emotions studied for people with ADHD. It could be that people with ADHD experience both emotional dysregulation for positive and negative emotions, but that the behavior leading from positive emotions is not seen as problematic by the people with ADHD and therefore did not give rise to interference in our version of the emotion Stroop task.

### 4.3 Research question 3: Stroop task for daily life domains in ADHD

The third research question focused on the application of a modified Stroop task in five daily life domains: work and study, relationships, social connections, free time, and self-image. At the group level, the results suggest that the current set up could be used to distinguish ADHD from control subjects, but with a small effect. Of the five daily life domains, only the social domain had predictive value in the logistic regression. At the individual level a small set of topics was pinpointed per person out of the potential 50 words. Further study in a clinical setting should investigate whether these few topics are indeed the most important problems for a participant.

Previous literature suggests a close tie between social anxiety and ADHD. In a study by Edel et al. (2010) 40% of the participants diagnosed with ADHD, met the criteria for social anxiety. At least 70% of these participants had already received therapy since they were on medication. So even after having received help, social anxiety was at a diagnostic level. Similar results were found by Koyuncu et al. (2019) who selected people with social anxiety and found that 60% of the participants had an ADHD diagnosis. Furthermore, it was found in a review on anxious children, that they showed impairment in both emotional processing and attentional control and that anxious children had a slowed response to faces irrespective of the emotional valence of the faces (Morellini et al., 2022). This could indicate that children with ADHD are in general more anxious. Follow-up studies with more focus on the social aspects and aspects of anxiety in ADHD are therefore recommended.

Another possible explanation for this finding might be the COVID lockdown at the time of data collection and the social distancing regulations, potentially resulting in exacerbated social issues. In a study among people with ADHD, it was found that 41% reported problems with social isolation during COVID (Sibley et al., 2021).

A reason for not finding significant results for the other four domains could be that the other four domains might have been ill defined. Also, it could be that our selection of words in the other four domains did not encompass the most difficult problems. Further research into word lists might give insight here.

### 4.4 Recommendations for therapists

We developed our application to support therapist in finding out what someone’s personal issues are in a quick and objective way. We aimed for this analysis to be done before a therapeutic visit. As this was only a proof of concept study, we did not yet manage to test whether this application actually points out the most important issues that need addressing first. Somewhat promising, our application did identify a small set of issues for each participant. Moreover, our ADHD personality trait Stroop task and the emotion Stroop task both resulted in findings as expected, therefore our modified daily life Stroop task is expected to work in the same way, and might therefore be useful for helping the therapist and patient.

The ADHD daily life task could be used after diagnosis, to see with which topic to start that day’s therapy session. Moreover, the test could be repeated throughout the sessions to see if new, or other, issues have arisen. Also, it could be an instrument to check if progress has been made with respect to something that was previously an issue.

Furthermore, the ADHD daily life Stroop task could potentially be used without a diagnosis. Although this might seem interesting, most probably the diagnosis would become clear to the therapist by the set of issues that a person struggles with. However, it could be worthwhile to start help immediately and then find out about a diagnosis in the process.

Furthermore, this application could help with disentangling comorbidity with other disorders. Often people with ADHD have other mental issues at the same time, like depression, social anxiety, autism, or bipolar disorder. With this application it might be possible to find out which disorder should be approached first to support the patient the most. In that case new word lists should be created that disentangle issues related to each separate disorder to see which one is triggered the most at a specific point in time. At this point it is unclear whether the daily life Stroop task would also work for other disorders, but since it works for associations like in the study by Sparrow et al. (2011), and for all sorts of concerns as shown by Williams et al. (1996), it might be possible to create similar word lists that work for other disorders.

A more risky application could be to offer the application to people to use in their home-setting by themselves. In that case people could take the Modified Daily Life Stroop task and find out which topics they struggle with at any point in time. In response to this, they could track their own issues and potentially become aware of issues that remain an issue for them over a long period. This could then be a trigger for people to find professional help or social support to work on that problem. Insight into one’s own mental health could empower our society in monitoring one’s mental state without immediate need for professional help to find out what is the problem someone is struggling with.

## 5 Conclusion

The current study shows that there are significant differences between subjects with ADHD and controls, especially in negative stimuli. A clinical study needs to be done to validate that the words of interest from the experiment are comparable to the daily life issues that are most urgent for that individual. However, the design of the modified Stroop task shows great potential to give support to the therapist by digitalizing the analysis of daily life issues in people with ADHD and freeing up valuable time in the therapy treatment. Moreover, this application could deliver a more personally attuned therapy session. Furthermore, it might offer the therapist an objective way to track the improvements in the patient throughout the therapy treatment.

Additionally, even though this was not the goal of this study, the application also shows to have some diagnostic value, which could prove to be an objective contribution to the current mostly subjective diagnostic procedure for ADHD.

Finally, this application could prove to be a valuable instrument for research on ADHD in general. Throughout the duration of the therapy the patients could be tracked. Thereby delivering an objective measure throughout the therapy to see how different problems change over time and which are most prevalent in the entire ADHD population. Here, we saw that social issues were the most prominent problem among our participants with ADHD. Similarly, we saw that negative emotions cause a lot of cognitive dissonance compared to controls, showing that our society, designed for neurotypicals, might be tough with respect to negative stimuli and social demands for people with ADHD.

## Data Availability

The datasets presented in this article are not readily available because the answers of participants are medical data and therefore personal data, which is not openly shared for other studies or other researchers besides the authors of this work. However, data on the method, word-lists or analysis are shared openly. Requests to access the datasets should be directed to s.schoenmakers@tue.nl.
