# Supplementary material for: Developing a therapeutic app based on the emotional Stroop task for objective discovery of daily life issues for people with ADHD
Source: Front Psychol. 2025 Mar 12;16:1502914. doi: 10.3389/fpsyg.2025.1502914 (PMC11936914; doi:10.3389/fpsyg.2025.1502914)
Supplement: Supplementary file 1 [file Data_Sheet_1.pdf]

1 SUPPLEMENTARY

of "Developing a therapeutic app based on the emotional Stroop task for objective discovery of daily life issues for people with ADHD" by S. Schoenmakers, S. H. Bos and W. A. Ijsselsteijn

A MAIN EXPERIMENT STIMULI

**target**		**translation**	
een	zes	one	six
twee	zeven	two	seven
drie	acht	three	eight
vier	negen	four	nine
vijf	tien	five	ten

Table A1. Practice stimuli

**Dutch**			**translation**		
**target**	**unrelated**	**distraction**	**target**	**unrelated**	**distraction**
onrustig	zorgzaam	vierkant	restless	caring	square
spontaan	tevreden	schuiven	spontaneous	satisfied	shove
prikkelbaar	sympatiek	schaal	irritable	friendly	disk
nieuwsgierig	standvastig	regulier	curious	steadfast	regular
gefrustreerd	praktisch	nummer	frustrated	practical	number
eerlijk	ijverig	loodrecht	honest	dilligent	straight
beweeglijk	humeurig	dagelijks	fidgety	moody	daily
betrouwbaar	flexibel	bladzijde	reliable	flexible	page
aandachtig	contactloos		attentive	unconnected	
zelfverzekerd	kieskeurig		confident	picky	
vol hoofd	imiterend		full head	copycat	
rusteloos	grappig		jumpy	funny	
onderbrekend	gehoorzaam		interrupting	obedient	
moedig	doorzettend		brave	persistent	
impulsief	beschermend		impulsive	protecting	
enthousiast	behendig		enthusiastic	agile	
energiek	achterdochtig		energetic	suspicious	
creatief	aardig		creative	kind	
chaotisch	aantrekkelijk		chaotic	attractive	
avontuurlijk	aanhankelijk		adventurous	affectionate	

Table A2. Stimuli ADHD Section

negative	neutral	positive	distraction
woedend	ontspannen	opgewekt	zitplaats
walging	onbewust	vrolijk	respect
verdrietig	neutraal	verheugd	microscoop
ongeduldig	ingetogen	tevreden	antwoord
haat	gerustgesteld	opgewonden	
gefrustreerd	afwachtend	gelukkig	
angst	acceptatie	enthousiast	
agressief		dolblij	
**translation**			
furious	relaxed	joyful	seating
loathing	unconscious	cheerful	respect
sad	neutral	delighted	microscope
impatient	modest	satisfied	answer
hate	reassured	excited	
frustrated	anticipating	happy	
fear	acceptance	enthusiastic	
agression		overjoyed	

Table A3. Stimuli Emotion Section

Domain 1:	Work		translation		
**target**	**unrelated**	**distraction**	**target**	**unrelated**	**distraction**
concentratie	taak	plastic	concentrate	task	plastic
veel tegelijk	student	plafond	many at the same time	student	ceiling
te laat komen	proactief	kleiner	be late	proactive	smaller
overwerkt	mail	adres	burnout	email	address
mislukken	loopbaan		fail	career	
hyperfocus	ijverig		hyperfocus	zealous	
details vergeten	computer		forget details	computer	
deadlines	collega		deadlines	colleague	
belemmerd	bureau		obstructive	desk	
afgeleid	arbeid		distracted	labor	
**Domain 2:**	**Relationships & Family**		**translation**		
**target**	**unrelated**	**distraction**	**target**	**unrelated**	**distraction**
sleur	vriendschap	toestel	rut	friendship	device
uitgekeken	spelen	schelen	disinterested	playing	differ
ruzie	samen zijn	kronkel	argument	together	winding
rotzooi	relatie	globaal	mess	relation	globally
ongeorganiseerd	partner		unorganised	partner	
onbedoeld	ouders		unintended	parents	
impulsief	liefde		impulsive	love	
bindingsangst	gesprekken		fear of commitment	conversations	
afwijzing	familie		rejection	family	
aandachtstekort	activiteiten		attention deficit	activities	
**Domain 3:**	**Social Connections**		**translation**		
**target**	**unrelated**	**distraction**	**target**	**unrelated**	**distraction**
confrontatie	vriendschap	steiger	confrontation	friendship	scaffold
uitgesproken	vriendenkring	schroef	pronounced	social circle	screw
teveel praten	netwerk	pamflet	talkative	network	flyer
pesten	maatschappij	grendel	bullying	society	hinge
onderhouden	gezelschap		upkeep	company	
onderbreken	gezellig		interrupt	pleasant	
onbegrip	feest		misinterpretation	party	
buitengesloten	borrelen		excluded	have drinks	
afwijzing	bezoeken		rejection	visit	
afgeleid	behulpzaam		distracted	helpful	
**Domain 4:**	**Hobby & Free Time**		**translation**		
**target**	**unrelated**	**distraction**	**target**	**unrelated**	**distraction**
niet ontspannen	weekend	textiel	not relaxed	weekend	textile
veel uitgeven	verzamelen	schrift	spend a lot	collect	notebook
sporten	vakantie	rubriek	sports	vacantion	section
risico's	tijdsverdrijf	ijskast	risks	pastime	freezer
hoofd legen	televisie		empty head	television	
fanatiek	reizen		fanatic	travel	
energie kwijt	liefhebber		exhausted	admirer	
boetes	lezen		fines	reading	
blessures	interessant		injuries	interesting	
actief	amateur		active	amateur	
**Domain 5:**	**Self image**		**translation**		
**target**	**unrelated**	**distraction**	**target**	**unrelated**	**distraction**
onzeker	zelfkennis	sleutel	insecure	know thyself	key
presenteren	spiegel	schotel	present	mirror	dish
perfectie	minderwaardig	embleem	perfection	inferior	emblem
oordeel	lelijk	drempel	judgement	ugly	threshold
onbegrip	karakter		misunderstanding	character	
mislukken	imago		fail	image	
kritiek	eigenwaarde		criticism	confidence	
faalangst	eigendunk		fear of failure	self esteem	
explosief	ego		explosive	ego	
burn-out	belangrijk		burn-out	important	

Table A4. Stimuli Daily Life section
